# Protein Kinase G is a key regulator of murine white adipogenesis

**DOI:** 10.1186/1471-2210-11-S1-P48

**Published:** 2011-08-01

**Authors:** Michaela M Mitschke, Daniela Scholz, Katrin Zimmermann, Peter Mayer, Bodo Haas, Alexander Pfeifer, Ana Kilic

**Affiliations:** 1 Institute of Pharmacology and Toxicology, University of Bonn, 53105 Bonn, Germany; 2 Pharma-Center, University of Bonn, 53113 Bonn, Germany; 3 Federal Institute for Drugs and Medical Devices, 53175 Bonn, Germany

## Background

Obesity, the excessive accumulation of adipose tissue, is the result of an imbalance in energy intake and output. Mammals posses two functionally distinct types of adipose tissue, namely white adipose tissue (WAT) and brown adipose tissue (BAT). In addition to its primary role for energy storage, WAT is a hormone and cytokine secreting organ involved in inflammatory responses. In contrast, BAT dissipates energy. Protein kinase G (PKGI) has been shown to be indispensable for smooth muscle cell relaxation and recently, we have demonstrated a crucial role for PKGI in BAT. In the present study, we investigated the role of PKGI in white adipocyte differentiation.

## Materials and results

PKGI is expressed in WAT throughout differentiation (i.e. in preadipocytes, differentiated adipocytes and fat tissue) as demonstrated by both RT-PCR and Western blot analysis. Differentiation of PKGI^fl/fl^ preadipocytes resulted in a pronounced lipid accumulation as evidenced by Oil Red O staining. In contrast, PKGI^fl/fl^ preadipocytes infected with LV-Cre (PKGI^0/0^) exhibited reduced differentiation. Analysis of triglyceride content revealed a significant decrease of TG levels by 65% ± 1% in PKGI^0/0^ as compared to PKGI^fl/fl^ adipocytes. Western blot analysis of adipose marker in white adipocytes showed a significant decrease of C/EBPα (19% ± 4.8%), PPARγ (66% ± 2.9%) and aP2 (37% ± 10.6%) expression in PKGI^0/0^ cells as compared to PKGI^fl/fl^. Supporting evidence is provided by a gain-of-function approach based on lentiviral overexpression of PKGI in 3T3-L1 cells (LV-PKGI) and stimulation by the 8-pCPT-cGMP, which resulted in increased fat accumulation and enhanced adipose marker expression.

Investigating the underlying PKG-dependent signalling mechanism, we found that LV-PKGI 3T3-L1 cells show a significant increase in RhoA Ser-188 phosphorylation and a coincidental decrease IRS-1 Ser-636/639 phosphorylation upon 8-pCPT-cGMP stimulation compared to wt cells. These results suggest that PGKI mediates the increase of adipogenesis by affecting RhoA/ROCK as well as Insulin signaling.

## Conclusion

Taken together, our data show that PKGI is a key player in white adipocyte differentiation.

